# An Incidental Finding of Congenital Complete Heart Block Presenting in Active Labor: A Multidisciplinary Approach

**DOI:** 10.7759/cureus.23393

**Published:** 2022-03-22

**Authors:** Bikash B Bora, Sanjib Baruah, Alokjyoti Malakar, Sandeep Dey, Pankaj Baruah, Ibharani Morang

**Affiliations:** 1 Anaesthesiology, Sanjivani Hospital, Jorhat, IND; 2 Anaesthesiology, Jorhat Christian Medical Centre, Jorhat, IND; 3 Cardiology, Jorhat Medical College and Hospital, Jorhat, IND; 4 Anaesthesiology, Jorhat Medical College and Hospital, Jorhat, IND; 5 Obstetrics and Gynaecology, Sanjivani Hospital, Jorhat, IND; 6 Obstetrics and Gynaecology, Jorhat Christian Medical Centre, Jorhat, IND

**Keywords:** spinal anesthesia, temporary pacemaker, permanent pacemaker implantation (ppm), high-risk pregnancy, pregnancy and heart disease, congenital complete heart block

## Abstract

Congenital complete heart block is a rare occurrence. In some cases, it remains asymptomatic until adulthood or in the case of women until pregnancy. It is usually secondary to placental transfer of maternal antibodies and is associated with high mortality and morbidity. We present a case of a parturient who presented in active labor with premature rupture of membranes and decreased fetal movements. We found that the patient had a complete heart block with mild effort intolerance on evaluation. Markers for metabolic and ischemic causes were negative, and we made a provisional diagnosis of congenital complete heart block. The patient underwent a lower section cesarian section under spinal anesthesia with temporary pacemaker backup. Postoperatively, the patient underwent permanent pacemaker implantation. This case report underlines the importance of standard American Society of Anesthesiologists (ASA) monitoring, including a 12-lead electrocardiogram (ECG), which could prove decisive and life-saving in dire circumstances.

## Introduction

Congenital complete heart block (CHB) is a rare condition. The estimated incidence described in the literature is one in 15,000 - 20,000 live births [1]. It may be associated with congenital structural heart disease [2] or neonatal lupus, secondary to the fetoplacental transfer of maternal anti-Ro/La or ribonucleoprotein (RNP) autoantibodies [3]. Rarely, in 30% of cases, it may remain asymptomatic and detected for the first time during adulthood or, in the case of women, during pregnancy [4]. Owing to the benign nature of the disease in a significant proportion of the population and the risk of associated mortality and morbidity among parturients, a high degree of suspicion and an integrated approach involving different disciplines are necessary for safe patient outcomes.

## Case presentation

**Figure 1:**
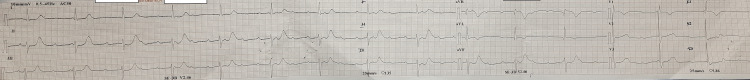
ECG showing CHB with a narrow QRS complex and severe bradycardia ECG: electrocardiogram; CHB: complete heart block

A 31-year-old parturient (parity 1) presented to the casualty at 37 weeks of gestation in active labor with premature rupture of membranes and decreased fetal movements. On evaluation, the patient was severely anemic (hemoglobin: 6.8 g%), had severe bradycardia (35 beats/minute), and class I - II effort intolerance. There was no history of any concomitant illness during her pregnancy or past. Other general and systemic examinations were unremarkable, except for bilateral pitting pedal edema (since the seventh month of gestation). A 12-lead ECG revealed a CHB (Figure 1). We took a cardiology opinion, and considering the urgent need for delivery, we planned an emergency cesarean section under spinal anesthesia with temporary pacemaker implantation (TPI).

**Figure 2:**
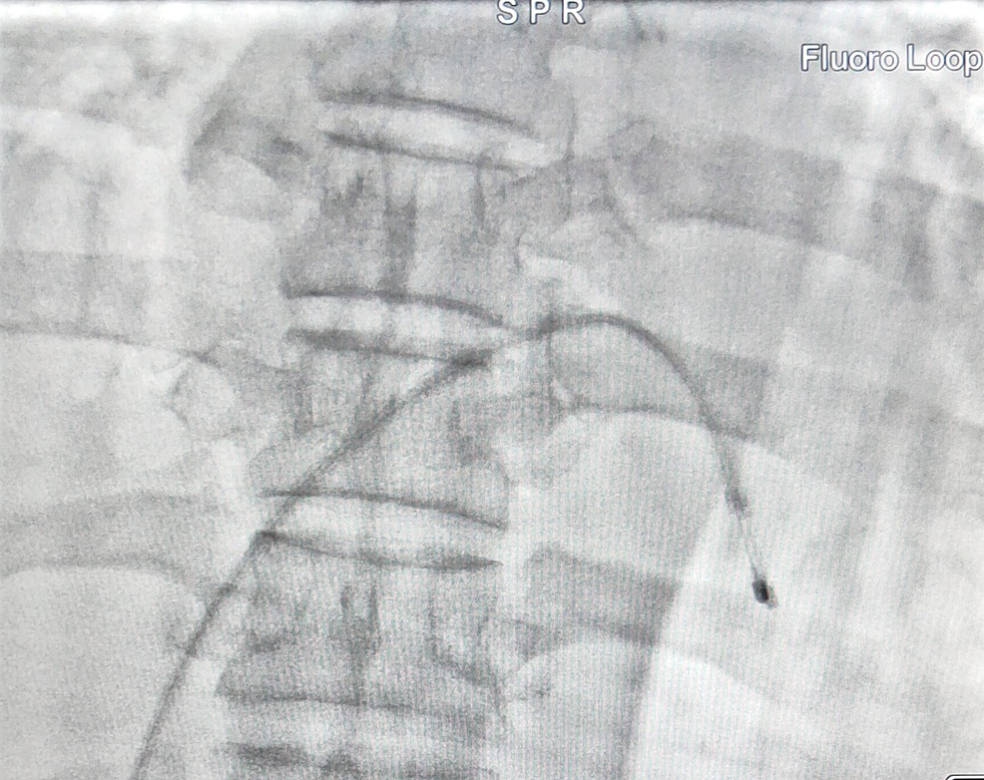
Fluoroscopic image showing temporary pacing lead at RV apex RV: right ventricle

In the operating room, we attached standard ASA monitors to the patient. We inserted temporary pacing via the right femoral vein under fluoroscopic view (approximately 30 seconds of fluoroscopic exposure was needed, at the chest level and with the lower back and abdomen of the patient covered by a lead shied) (Figure 2). We set the pacing rate at 60 beats per minute. The patient was then positioned in the left lateral position with the left lower limb flexed and the right lower limb extended, intending to avoid displacement of the pacing lead. Spinal anesthesia (subarachnoid block) was administered with 10 mg bupivacaine heavy and 20 µg fentanyl in lumber 3-4 interspace using 25G Quincke’s needle. The pinprick method confirmed the onset of sensory block up to thoracic 6 level. The obstetric team delivered a 2.9 kg healthy baby with an APGAR (Appearance, Pulse, Grimace, Activity, and Respiration) score of 10/10 following a lower section Pfannenstiel and midline laparotomy incisions. There was uterine atony following delivery. Atony of the uterus was managed with a supplemental dose of 600 mcg misoprostol rectally and 200 mcg methylergometrine intramuscularly, and 100 mcg carbetocin given intravenously immediately post-delivery. The patient received 1000 ml Ringer’s lactate and one unit of packed cell volume (PCV) during the intraoperative period. After surgery, the patient was shifted to the post-anesthesia care unit and then transferred to the intensive care unit (ICU) for continuous monitoring and further management.

A cardiology team evaluated the patient in the ICU. The patient had mild symptoms (class I - II effort intolerance) without syncope or palpitation. Transthoracic echocardiography (ECHO) was consistent with mild concentric left ventricular hypertrophy, an enlarged left atrium, mild tricuspid regurgitation, trivial mitral and aortic regurgitation, normal left ventricular systolic function, and a left ventricular ejection fraction of 69%. Screening for collagen vascular disorder by the antinuclear antibody was negative. Markers for ischemic heart disease like serum myoglobin, cardiac troponin-T, cardiac troponin-I, and creatinine kinase myocardial band were also within the normal range. Neither was there any sign of regional wall motion abnormality on ECHO. Based on the above clinical findings, the paucity of cardiac symptoms, and the presence of a narrow QRS complex with CHB, we made a provisional diagnosis of congenital CHB.

The patient was under continuous cardiac monitoring during recovery, and we kept the TPI as a backup (pacing rate lowered and kept sensing). The patient received three units of PCV transfusions postoperatively to optimize her hemoglobin status. During the following 96-hour observation period, we did not observe any episode of asystole. Subsequently, the patient had undergone permanent pacemaker implantation (PPI).

## Discussion

CHB can be congenital or acquired. Acquired CHB is rare in pregnancy, and the usual diagnosis is after the fifth decade of life [5]. During pregnancy, acquired CHB has been described as secondary to systemic lupus erythematosus, valvular heart disease, or ischemic heart disease [6-8]. Other possible causes of acquired CHB in pregnancy include atrial stretch secondary to increased plasma volume and the effect of estrogen on cardiac myocytes [9,10]. In our case, markers for collagen vascular disease and ischemic heart disease were negative, and there were no significant structural or valvular heart disease on ECHO. The findings of ventricular hypertrophy and mid to trivial regurgitation are consistent with regular physiological changes during pregnancy [11].

The literature has described that a complete congenital heart block affects 15,000 - 20,000 live births [1]. It may be associated with complex structural heart disease like heterotaxy syndrome. Such cases are associated with fetal mortality, even with pacing [12-14]. CHB can also be secondary to exposure to maternal Ro/La autoantibodies in early fetal life. Such patients usually require pacing early in life and have associated high mortality [15]. Congenital CHB can also lead to sudden cardiac arrest in patients with wide QRS complex on ECG [16] or those associated with complex structural heart disease [12-14]. In our case, we have decided to go forward with the case under temporary pacing. Our decision for temporary pacing was because this patient had a complete third-degree atrioventricular block with minimal symptoms with severe bradycardia (heart rate of approximately 34 beats per minute), which was for perioperative backup [17-19].

Approaches to temporary pacing can be multifold. It may be transvenous, endocardial pacing (internal and external jugular, subclavian, brachial, or femoral) [20], or epicardial pacing (transcutaneous or transesophageal) [21,22]. In our case, we opted for temporary pacing via a transfemoral route under fluoroscopic guidance. We used fluoroscopy as the fetus was far beyond the period of radiosensitivity [23], ease of the procedure, and individual expertise.

In the absence of any obstetric indication, existing literature describes vaginal delivery as the safest mode of delivery [24]. In cases that demand cesarian section for emergent termination of pregnancy, both regional (graded epidural or low-dose combined spinal-epidural) and general anesthesia have been described as safe and effective, provided bradycardia and hypotension are avoided [24,25]. It is imperative to avoid high spinal [26], use of drugs like succinylcholine and fentanyl, and keep an emergency backup of rescue agents like atropine or isoproterenol in the absence of temporary or permanent pacing [27]. In our case, we opted for low-dose spinal anesthesia under TPI backup as the patient was in labor and due to technical difficulty in placing the epidural catheter without dislodging the pacing lead. As the patient had a CHB (likely to be congenital) without any other modifiable cause like metabolic or ischemic, we advised the patient to opt for a permanent pacemaker.

## Conclusions

Congenital CHB, although rare in adults and especially in pregnant women, has tremendous prognostic implications, given the high risk of mortality and morbidity associated with it, both to the mother and the fetus. The management of CHB poses a more significant challenge when diagnosed during late pregnancy or when a patient has already gone into labor. This case report stresses the importance of standards of essential ASA monitoring, such as, and not limited to, continuous pulse rate and ECG monitoring. The high degree of vigilance, stressing upon the basic standards laid down by ASA, and expertise in minimum life-saving skills can help prevent major complications and save a life.

## References

[1] Mandal S, Mandal D, Sarkar A, Biswas J, Panja M (2015). Complete heart block and pregnancy outcome: an analysis from Eastern India. SOJ Gynecol Obstet Womens Health.

[2] Kertesz NJ, Fenrich AL, Friedman RA (1997). Congenital complete atrioventricular block. Tex Heart Inst J.

[3] Friedman DM, Rupel A, Glickstein J, Buyon JP (2002). Congenital heart block in neonatal lupus: the pediatric cardiologist's perspective. Indian J Pediatr.

[4] Reid JM, Coleman EN, Doig W (1982). Complete congenital heart block. Report of 35 cases. Br Heart J.

[5] Perloff JK (2003). Clinical Recognition of Congenital Heart Disease. Fifth Edition. https://pesquisa.bvsalud.org/portal/resource/pt/dan-2617?lang=en.

[6] Tateno S, Niwa K, Nakazawa M, Akagi T, Shinohara T, Yasuda T (2003). Arrhythmia and conduction disturbances in patients with congenital heart disease during pregnancy: multicenter study. Circ J.

[7] Suri V, Keepanasseril A, Aggarwal N, Vijayvergiya R, Chopra S, Rohilla M (2009). Maternal complete heart block in pregnancy: analysis of four cases and review of management. J Obstet Gynaecol Res.

[8] Lo CH, Wei JC, Tsai CF, Li LC, Huang SW, Su CH (2018). Syncope caused by complete heart block and ventricular arrhythmia as early manifestation of systemic lupus erythematosus in a pregnant patient: a case report. Lupus.

[9] Eddy WA, Frankenfeld RH (1977). Congenital complete heart block in pregnancy. Am J Obstet Gynecol.

[10] Machuki JO, Zhang HY, Harding SE, Sun H (2018). Molecular pathways of oestrogen receptors and β-adrenergic receptors in cardiac cells: Recognition of their similarities, interactions and therapeutic value. Acta Physiol.

[11] Sanghavi M, Rutherford JD (2014). Cardiovascular physiology of pregnancy. Circulation.

[12] Nakamura FF, Nadas AS (1964). Complete heart block in infants and children. N Engl J Med.

[13] Schmidt KG, Ulmer HE, Silverman NH, Kleinman CS, Copel JA (1991). Perinatal outcome of fetal complete atrioventricular block: a multicenter experience. J Am Coll Cardiol.

[14] Michaëlsson M, Jonzon A, Riesenfeld T (1995). Isolated congenital complete atrioventricular block in adult life. A prospective study. Circulation.

[15] Villain E, Coastedoat-Chalumeau N, Marijon E, Boudjemline Y, Piette JC, Bonnet D (2006). Presentation and prognosis of complete atrioventricular block in childhood, according to maternal antibody status. J Am Coll Cardiol.

[16] Molthan ME, Miller RA, Hastreiter AR, Paul MH (1962). Congenital heart block with fatal Adams-Stokes attacks in childhood. Pediatrics.

[17] Bob-Manuel T, Nanda A, Latham S, Pour-Ghaz I, Skelton WP IV, Khouzam RN (2018). Permanent pacemaker insertion in patients with conduction abnormalities post transcatheter aortic valve replacement: a review and proposed guidelines. Ann Transl Med.

[18] Kosztin A, Boros AM, Geller L, Merkely B (2018). Cardiac resynchronisation therapy: current benefits and pitfalls. Kardiol Pol.

[19] Samii SM (2015). Indications for pacemakers, implantable cardioverter-defibrillator and cardiac resynchronization devices. Med Clin North Am.

[20] (1993). Choice of route for insertion of temporary pacing wires: recommendations of the Medical Practice Committee and Council of the British Cardiac Society. Br Heart J.

[21] Zoll PM (1952). Resuscitation of the heart in ventricular standstill by external electric stimulation. N Engl J Med.

[22] Santini M, Ansalone G, Cacciatore G, Turitto G (1990). Transesophageal pacing. Pacing Clin Electrophysiol.

[23] Dauer LT, Thornton RH, Miller DL (2012). Radiation management for interventions using fluoroscopic or computed tomographic guidance during pregnancy: a joint guideline of the Society of Interventional Radiology and the Cardiovascular and Interventional Radiological Society of Europe with Endorsement by the Canadian Interventional Radiology Association. J Vasc Interv Radiol.

[24] Baghel K, Mohsin Z, Singh S, Kumar S, Ozair M (2016). Pregnancy with complete heart block. J Obstet Gynaecol India.

[25] George M, Kurian D, Salim SV (2020). Spinal anaesthesia for cesarean section in a case of congenital complete heart block. J Obstet Anaesth Crit Care.

[26] Modi MP, Butala B, Shah VR (2006). Anaesthetic management of an unusual case of complete heart block for LSCS. Indian J Anaesth.

[27] Agrawal A, Guzman DB (2018). Third-Degree Atrioventricular Block (Complete Heart Block) Medication. https://emedicine.medscape.com/article/162007-medication.

